# Supplementary material for: Local ancestry inference with poorly-matched reference panels
Source: PLoS Genet. 2026 Jul 13;22(7):e1011919. doi: 10.1371/journal.pgen.1011919 (PMC13375125; doi:10.1371/journal.pgen.1011919)
Supplement: S1 Appendix — (PDF) [file pgen.1011919.s001.pdf]

S1 Appendix. Estimation of switch rate

At the clustering step, when we create the initial model file, we need estimates of the ancestry-specific switch rates. Let ρ_i be the rate of switching between copied haplotypes FLARE's model [3] when the ancestry is *i.*

First, in the pre-clustering FLARE step, for each admixed haplotype *k* and marker *m*, we obtain an estimate of

$$\tau_{m,k} = d_m \rho_{m,k}$$

where d_m is the distance in Morgans between markers *m* and *m+1,* and $\rho_{m,k}$ is the switch rate between markers *m* and *m+1* for admixed haplotype *k*, using the method described for Beagle's haplotype phasing [22]. We sum this across marker intervals in the window w, and divide by the total genetic distance to obtain an estimate of $\rho_{w,k}$, the switch rate for admixed haplotype *k* in window *w*.

After performing clustering, we assign each haplotype/window pair to its most likely ancestry $a_{w,k}$, and we obtain the ancestry-specific switch rate as

$$\hat{\rho}_i = \frac{\sum_{w,k} \hat{\rho}_{w,k} 1\{a_{w,k} = i\}}{\sum_{w,k} 1\{a_{w,k} = i\}}$$

where $1\{a_{w,k} = i\}$ is one if the assigned ancestry for haplotype *k* in window *w* is *i*, and zero otherwise.
