# Supplementary material for: Local ancestry inference with poorly-matched reference panels
Source: PLoS Genet. 2026 Jul 13;22(7):e1011919. doi: 10.1371/journal.pgen.1011919 (PMC13375125; doi:10.1371/journal.pgen.1011919)
Supplement: S2 Appendix — (PDF) [file pgen.1011919.s002.pdf]

S2 Appendix. Updated estimates of ancestry-specific copying probabilities *P*

In the original FLARE, the matrix of copying probabilities is determined in the initialization step and is not updated in the EM steps [3]. When performing the third/final step of clustered FLARE, we start with the copying matrix estimated in the clustering step, and update it in the EM steps using the equation given below.

We denote the (i,*j*)-th entry of *P* as $p_{j|i}$ which is the probability that the FLARE model copies from the *j*-th reference panel when the ancestry is *i*. The posterior probability for state (*i*, *h*) for ancestry *i* and reference haplotype *h* at marker *m* is proportional to the product of the forward and backward probabilities $\alpha_{ih}(m) \times \beta_{ih}(m)$. Thus,

$$\hat{p}_{j|i} = \sum_{k}\sum_{m}\sum_{h \text{ in panel } j} \alpha_{ih}(m) \times \beta_{ih}(m) \Big/ \sum_{k}\sum_{m}\sum_{h} \alpha_{ih}(m) \times \beta_{ih}(m),$$

where *k* indexes the admixed samples.
