# Supplementary material for: Local ancestry inference with poorly-matched reference panels
Source: PLoS Genet. 2026 Jul 13;22(7):e1011919. doi: 10.1371/journal.pgen.1011919 (PMC13375125; doi:10.1371/journal.pgen.1011919)
Supplement: S3 Appendix — (PDF) [file pgen.1011919.s003.pdf]

S3 Appendix. Simulation code

```
# This code was run as: python3 sim.py 100000000 1000 $seed $output_file_prefix
# with seeds 1, 2, and 3
# "AmericanAdmixture_4B11" from stdpopsim source code modified to add additional populations
long_description = """
    Demographic model for American admixture, taken from Browning et al. 2018.
    This model extends the Gravel et al. (2011) model of African/European/Asian
    demographic history to simulate an admixed population with admixture
    occurring 12 generations ago. The admixed population had an initial size
    of 30,000 and grew at a rate of 5% per generation, with 1/6 of the
    population of African ancestry, 1/3 European, and 1/2 Asian. Note that this
    demographic model was not inferred, and the mutation rate that Browning et al.
    used for simulation is smaller than used for inferring the model,
    so the mutation rate provided here is that from Gravel et al.
  """

import math, msprime, sys, gzip
import numpy as np

genomelen = int(sys.argv[1])
nsamp = int(sys.argv[2]) # samples per population
seed = int(sys.argv[3])
outprefix = sys.argv[4]

populations = [
    dict(name="AFR0", description="Contemporary African population"),
    dict(name="EUR0", description="Contemporary European population"),
    dict(name="ASIA0", description="Contemporary Asian population"),
    dict(name="ADMIX0", description="Modern 3-way admixed population"),
    dict(name="AFR2", description="Diverged from AFR by 600 generations"),
    dict(name="EUR2", description="Diverged from EUR by 600 generations"),
    dict(name="ASIA2", description="Diverged from ASIA by 600 generations"),
    dict(name="ADMIX1", description="Modern 2-way 20/80 EUR/ASN admixed population"),
    dict(name="ADMIX3", description="Modern older admixed population")
]
npops = len(populations)

mutation_rate = 2.36e-8
recomb_rate = 1e-8

N0 = 7310  # initial population size
Thum = 5920  # time (gens) of advent of modern humans
Naf = 14474  # size of african population
Tooa = 2040  # number of generations back to Out of Africa
```

```
Nb = 1861  # size of out of Africa population
mafb = 1.5e-4  # migration rate Africa and Out-of-Africa
Teu = 920  # number generations back to Asia-Europe split
Neu = 1032  # bottleneck population sizes
Nas = 554
mafeu = 2.5e-5  # mig. rates
mafas = 7.8e-6
meuas = 3.11e-5
reu = 0.0038  # growth rate per generation in Europe
ras = 0.0048  # growth rate per generation in Asia
Tadmix = 12  # time of admixture for ADMIX0 and ADMIX1/2
Tadmixold = 50 # time of admixture for ADMIX3
Nadmix = 30000  # initial size of admixed population
radmix = 0.05  # growth rate of admixed population
# pop0 is Africa, pop1 is Europe, pop2 is Asia,  pop3 is admixed
Tdiv2 = 600 # time in gens of divergence to AFR2 EUR2 ASIA2

population_configurations = [
    msprime.PopulationConfiguration(
      initial_size=Naf, growth_rate=0.0, metadata=populations[0]
    ),
    msprime.PopulationConfiguration(
      initial_size=Neu * math.exp(reu * Teu),
      growth_rate=reu,
      metadata=populations[1],
    ),
    msprime.PopulationConfiguration(
      initial_size=Nas * math.exp(ras * Teu),
      growth_rate=ras,
      metadata=populations[2],
    ),
    msprime.PopulationConfiguration(
      initial_size=Nadmix * math.exp(radmix * Tadmix),
      growth_rate=radmix,
      metadata=populations[3],
    ),
    msprime.PopulationConfiguration(
      initial_size=Naf, growth_rate=0.0, metadata=populations[4]
    ),
    msprime.PopulationConfiguration(
      initial_size=Neu * math.exp(reu * Teu), growth_rate=reu, metadata=populations[5]
    ),
    msprime.PopulationConfiguration(
      initial_size=Nas * math.exp(ras * Teu), growth_rate=ras, metadata=populations[6]
```

```
        ),
        msprime.PopulationConfiguration(
            initial_size=Nadmix * math.exp(radmix * Tadmix),
            growth_rate=radmix,
            metadata=populations[7],
        ),
        msprime.PopulationConfiguration(
            initial_size=Nadmix * math.exp(radmix * Tadmixold),
            growth_rate=radmix,
            metadata=populations[8],
        )
]

migration_matrix = [
    [0, mafeu, mafas, 0, 0, 0, 0, 0, 0],
    [mafeu, 0, meuas, 0, 0, 0, 0, 0, 0],
    [mafas, meuas, 0, 0, 0, 0, 0, 0, 0],
    [0, 0, 0, 0, 0, 0, 0, 0, 0],
    [0]*9,
    [0]*9,
    [0]*9,
    [0]*9,
    [0]*9
]
# Admixture event, 1/6 Africa, 2/6 Europe, 3/6 Asia (pop 3)
# plus an admixture with 20% Eur 80% Asia (pop 7)
# and an older event matching the first one in proportions (pop 8)
admixture_event = [
    msprime.MassMigration(
        time=Tadmix, source=3, destination=0, proportion=1.0 / 6.0
    ),
    msprime.MassMigration(
        time=Tadmix, source=3, destination=1, proportion=2.0 / 5.0
    ),
    msprime.MassMigration(time=Tadmix, source=3, destination=2, proportion=1.0),
    msprime.MassMigration(time=Tadmix, source=7, destination=1, proportion=0.2),
    msprime.MassMigration(time=Tadmix, source=7, destination=2, proportion=1),
    msprime.MassMigration(
        time=Tadmixold, source=8, destination=0, proportion=1.0 / 6.0
    ),
    msprime.MassMigration(
        time=Tadmixold, source=8, destination=1, proportion=2.0 / 5.0
    ),
    msprime.MassMigration(time=Tadmixold, source=8, destination=2, proportion=1.0),
```

```
]
# Divergence of related populations, Tdiv2 gen ago
div_event = [
   msprime.MassMigration(time=Tdiv2, source=4, destination=0, proportion=1.0),
   msprime.MassMigration(time=Tdiv2, source=5, destination=1, proportion=1.0),
   msprime.MassMigration(time=Tdiv2, source=6, destination=2, proportion=1.0),
   ]
# Asia and Europe split
eu_event = [
   msprime.MigrationRateChange(time=Teu, rate=0.0),
   msprime.MassMigration(time=Teu, source=2, destination=1, proportion=1.0),
   msprime.PopulationParametersChange(
      time=Teu, initial_size=Nb, growth_rate=0.0, population_id=1
   ),
   msprime.MigrationRateChange(time=Teu, rate=mafb, matrix_index=(0, 1)),
   msprime.MigrationRateChange(time=Teu, rate=mafb, matrix_index=(1, 0)),
]
# Out of Africa event
ooa_event = [
   msprime.MigrationRateChange(time=Tooa, rate=0.0),
   msprime.MassMigration(time=Tooa, source=1, destination=0, proportion=1.0),
]
# initial population size
init_event = [
   msprime.PopulationParametersChange(time=Thum, initial_size=N0, population_id=0)
]

census_event = [msprime.CensusEvent(Tadmixold+1)]

demographic_events = admixture_event + census_event + div_event + eu_event + ooa_event + init_event

mydemography = msprime.Demography.from_old_style(population_configurations,
migration_matrix=migration_matrix, demographic_events=demographic_events)

mysamples = dict.fromkeys(range(npops),nsamp)
totalnsamples = nsamp*npops
myrecomb = msprime.RateMap.uniform(genomelen,recomb_rate)

mytrees =
msprime.sim_ancestry(mysamples,demography=mydemography,random_seed=seed,recombination_rat
e=myrecomb) # can add record_full_arg=True and record_migrations=True

# add the mutations
mts = msprime.sim_mutations(mytrees,rate=mutation_rate,random_seed=seed)
```

```
# output the vcf
with gzip.open(outprefix+"sim_gts.vcf.gz", "wt") as f:
    mts.write_vcf(f)

# determine ancestry of each haplotype
# makes use of the census that occured further back than the admix_time
def node_get_pop(tree,node,admix_time):
    # if the time of a node is < admix_time, it's population is found by tracing up the tree
    oldnode = node
    while tree.get_time(node) <= admix_time:
        oldnode = node
        node = tree.get_parent(node)
    thispop = tree.get_population(node) # provisional
    return thispop

# print out any other files that will be needed
sampfile = open(outprefix+"sample_map.txt","w")
for j in range(npops):
    for i in range(nsamp):
        k = j*nsamp + i
        print("tsk_"+str(k)+"\t"+populations[j]['name'],file=sampfile)
sampfile.close()
readme = open(outprefix+"README","w")
print("# simulated 3 ancestry scenario with 13 populations in total",file=readme)
print("genomelen",genomelen,file=readme)
print("nsamp",nsamp,file=readme)
print("seed",seed,file=readme)
readme.close()

# print out the local ancestry at the variant sites
ancfile = gzip.open(outprefix+"la_true.vcf.gz","wt")
treeseq = mts
ancfile.write('##fileformat=VCFv4.1\n##filedate=20131220\n##source="mysim"\n##FORMAT=<ID=GT,N
umber=1,Type=String,Description="Genotype">\n')
myline = "#CHROM\tPOS\tID\tREF\tALT\tQUAL\tFILTER\tINFO\tFORMAT"
for i in range(totalnsamples):
    myline += "\ttsk_"+str(i)
ancfile.write(myline+'\n')
for tree in treeseq.trees():
    for x in tree.sites():
        pos = x.position
        posstr = str(int(pos))
        myline = "1\t"+posstr+"\t.\t0\t1,2,3,4,5,6,7,8,9\t.\t.\t.\tGT"
```

```
for i in range(totalnsamples*2):
    anc = str(node_get_pop(tree,i,Tadmixold+0.001))
    if i % 2 == 0: mysep = "\t"
    else: mysep = "|"
    myline += mysep+anc
ancfile.write(myline+'\n')
```
