# Supplementary material for: Local ancestry inference with poorly-matched reference panels
Source: PLoS Genet. 2026 Jul 13;22(7):e1011919. doi: 10.1371/journal.pgen.1011919 (PMC13375125; doi:10.1371/journal.pgen.1011919)
Supplement: S1 Fig — (PDF) [file pgen.1011919.s004.pdf]

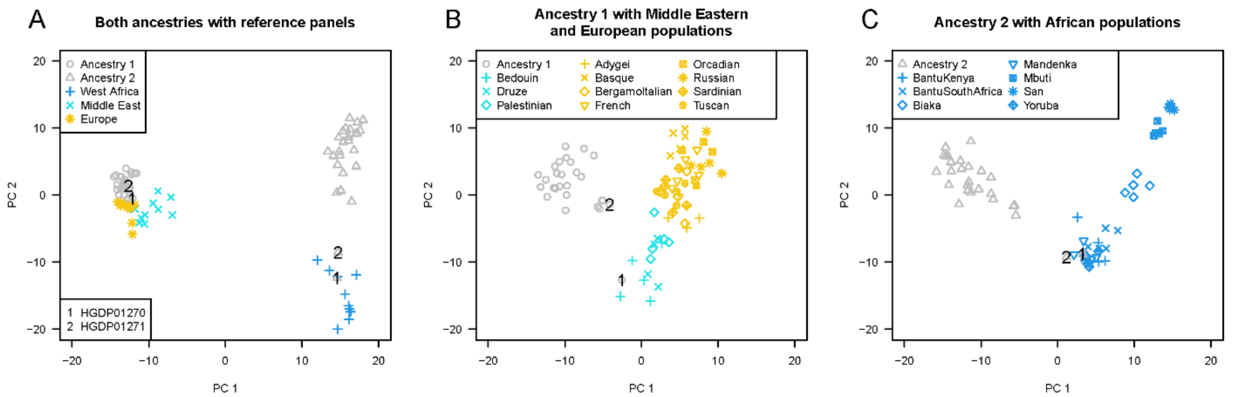

S1 Figure. Ancestry-specific multi-dimensional scaling for HGDP Mozabites with outliers notated. This figure is the same as Fig 4 in the main text, but with outliers notated. Each plot is from a separate multi-dimensional scaling analysis, which includes one or both Mozabite ancestries along with selected other individuals. The x and y-axes are the first two principal coordinates from each multi-dimensional scaling analysis. (A) Each Mozabite individual is represented by one gray circle (ancestry 1) and by one gray triangle (ancestry 2). Ten randomly selected individuals are included from each of the reference panels used in the local ancestry analysis: West Africa (Yoruba and Mandenka), Middle East (Druze), and Europe (French and Basque). (B) Ancestry 1 for the Mozabite individuals with five randomly selected individuals from each of the Middle Eastern (teal) and European (yellow) populations in the HGDP. (C) Ancestry 2 for the Mozabite individuals with 10 randomly selected individuals from each of the African (blue) populations in the HGDP. Two outlier Mozabite individuals are notated with the digits 1 and 2 in all plots.
