# Supplementary material for: Local ancestry inference with poorly-matched reference panels
Source: PLoS Genet. 2026 Jul 13;22(7):e1011919. doi: 10.1371/journal.pgen.1011919 (PMC13375125; doi:10.1371/journal.pgen.1011919)
Supplement: S1 Table — (PDF) [file pgen.1011919.s005.pdf]

S1 Table. Autocorrelation as a metric for detecting the presence of spurious ancestries in simulated data.

Target	Reference	True N. Anc.	Analysis N. Anc.	Minimum autocorrelation	
				50 ref.	1000 ref.
3way12	Afr + Eur + As	3	3	0.48	0.72
2way12	Afr + Eur + As	2	2	0.48	0.83
3way12	Afr + Eur	3	4	0.18	0.17
2way12	Afr + Eur + As	2	3	0.16	0.21
Asia	AfrP + EurP + AsP	1	2	0.11	0.13
Asia	Afr + Eur	1	2	0.11	0.16

Target samples are 1000 haplotypes of either a simulated three-way admixture with admixture occurring 12 generations (3way12; see Fig 2), a simulated 2-way admixture which is 20% European and 80% Asian with admixture occurring 12 generations ago (2way12; see Fig 2), or simulated unadmixed Asians (Asia). Reference panels are made up of simulated African (Afr), European (Eur), or Asian (As) individuals, or proxies for these (AfrP, EurP, AsP), with 50 samples of each (second to rightmost column) or 1000 samples of each (rightmost column). The true number of ancestries in the target is shown, along with the number of ancestries specified in the analysis. In the first two rows, the number of specified ancestries matches the true number of ancestries, while in the remaining rows, the number of specified ancestries is one more than the true number of ancestries. We calculate the autocorrelation at a one-window (0.5 cM) lag for each ancestry, and the minimum value is given. Each result is based on a single replicate simulation of one chromosome with a length of 100 Mb.
